# Reperfusion injury in STEMI: a double-edged sword

**DOI:** 10.1186/s43044-025-00683-7

**Published:** 2025-09-05

**Authors:** Krupa Sara Thomas, Divina Mariya Puthooran, Sudeep Edpuganti, Adi Lakshmi Reddem, Angela Jose, Subramanya Sri Mahesh Akula

**Affiliations:** 1 Department of Medicine, Faculty of Medicine, Tbilisi State Medical University, Tbilisi, Georgia; 2 Department of Medicine, School of Health Sciences, University of Georgia, Tbilisi, Georgia

**Keywords:** STEMI, Reperfusion injury, Primary percutaneous coronary intervention (PPCI), Oxidative stress, Mitochondrial dysfunction, No-reflow phenomenon, Artificial intelligence, Ischemia–reperfusion injury, Cardioprotection

## Abstract

**Background:**

ST-elevation myocardial infarction (STEMI) is a major cardiac event that requires rapid reperfusion therapy. The same reperfusion mechanism that minimizes infarct size and mortality may paradoxically exacerbate further cardiac damage—a condition known as reperfusion injury. Oxidative stress, calcium excess, mitochondrial malfunction, and programmed cell death mechanisms make myocardial dysfunction worse. Even with the best revascularization techniques, reperfusion damage still jeopardizes the long-term prognosis and myocardial healing.

**Methods:**

A thorough narrative review was carried out using some of the most well-known scientific databases, including ScienceDirect, PubMed, and Google Scholar. With an emphasis on pathophysiological causes, clinical manifestations, innovative biomarkers, imaging modalities, artificial intelligence applications, and developing treatment methods related to reperfusion injury, peer-reviewed publications published between 2015 and 2025 were highlighted.

**Main body:**

The review focuses on the molecular processes that underlie cardiac reperfusion injury, such as reactive oxygen species, calcium dysregulation, opening of the mitochondrial permeability transition pore, and several types of programmed cell death. Clinical syndromes such as myocardial stunning, coronary no-reflow, and intramyocardial hemorrhage are thoroughly studied—all of which lead to negative consequences like heart failure and left ventricular dysfunction. Cardiac magnetic resonance imaging along with coronary angiography and significant biomarkers like N-terminal proBNP and soluble ST2 aid in risk stratification and prognosis. In addition to mechanical techniques like ischemia postconditioning and remote ischemic conditioning, pharmacological treatments are also examined. Despite promising research findings, the majority of therapies have not yet proven consistently effective in extensive clinical studies. Consideration of sex-specific risk factors, medicines that target the mitochondria, tailored therapies, and the use of artificial intelligence for risk assessment and early diagnosis are some potential future avenues.

**Conclusion:**

Reperfusion damage continues to be a significant obstacle to the best possible recovery after STEMI, even with improvements in revascularization. The management of STEMI still relies heavily on early reperfusion, although adjuvant medicines that target reperfusion injury specifically are desperately needed. Molecular-targeted approaches, AI-driven risk assessment, and precision medicine advancements have the potential to reduce cardiac damage and enhance long-term outcomes for patients with STEMI.

## Background

ST-elevation myocardial infarction (STEMI) is a potentially lethal cardiovascular emergency that is marked by partial or total blockage of the coronary arteries [1]. Reduced systolic function, cardiac remodeling, and heart failure occur due to ischemia [2]. The European Society of Cardiology reports that there were approximately 5.8 million cases of ischemic Heart disease in 2019 with approximately 38 percent of deaths occurring in women and 44 percent in men. An important cause of death worldwide is the rising incidence of cardiovascular disorders, which are mostly found in developing countries [3].

Acute coronary syndromes (ACS) affect over 7 million people worldwide each year. STEMI represents roughly 30% of all ACS cases [3]. Modifiable risk factors for STEMI include smoking, metabolic syndrome, hypertension, and abnormal lipid levels, which could lead to dyslipidemia and cholesterol imbalance [4, 5]. The severity of the illness also differs with age [5]. STEMI often occurs in middle-aged and older adults. For example, the average age of a first myocardial infarction is ~65 years in men and 72 in women. According to StatPearls (last updated Oct 6, 2024), approximately 38% of hospital presentations for acute coronary syndrome in the USA are STEMI [3].

The American College of Cardiology Foundation (ACCF)/American Heart Association (AHA)/Society for Cardiovascular Angiography and Interventions (SCAI) recommendations advise early revascularization in patients with acute coronary syndrome to control myocardial damage [6]. Reperfusion with primary percutaneous coronary intervention (PPCI) helps unblock occluded arteries and restores coronary blood flow [7]. Both symptoms, as well as quality of life, are improved by this prompt intervention which prevents cell death and ischemia [8]. When PPCI is unavailable, fibrinolytic drugs are used as an alternative [7]. Though advantageous, reperfusion therapy may also paradoxically worsen cardiac damage, a condition known as reperfusion injury (RI). This might lead to complications such as no-reflow phenomenon, myocardial stunning, malignant arrhythmias, and heart failure. These disorders are triggered by damage to cardiomyocytes and macrophages, resulting in the release of reactive oxygen species (ROS). Dysregulated autophagy can trigger apoptotic pathways, leading to cell death [8–10]. The clinical methods used to evaluate reperfusion are electrocardiogram and angiography; however, they have limitations in terms of accuracy. Additionally, contrast-enhanced magnetic resonance imaging (MRI), when used days after myocardial infarction (MI), may be too late, and hence is limited in daily practice [11]. STEMI is one of the most life-threatening conditions that needs to be diagnosed and managed early to decrease mortality and improve recovery. Although reperfusion treatment has increased the survival rate and reduced infarct burden, additional myocardial injury may occur, and hence, recovery is not guaranteed.

This review aims to explore the paradox of reperfusion therapy in STEMI: a double-edged sword, highlighting the pathophysiological mechanisms underlying reperfusion injury, its clinical implications, and new approaches to its management and prevention.

## Pathophysiological mechanisms of myocardial infarction reperfusion injury

Numerous investigations have focused on the pathophysiological factors behind myocardial infarction reperfusion injury (MIRI). Numerous types of programmed cell death, including apoptosis and endoplasmic reticulum stress, are among the mechanisms involved. Additional factors include oxidative stress, intracellular calcium excess, problems with energy metabolism, and autophagy. These interconnected mechanisms have the potential to either directly or indirectly worsen cell death [12].

### Oxidative stress

Cell death and vascular endothelial dysfunction are significantly influenced by ROS. Oxidative stress, tissue damage, and an imbalance between the antioxidant and oxidation systems are all brought on by its overproduction. ROS levels surge, especially during reperfusion, due to mechanisms like increased xanthine oxidase formation and mitochondrial electron transport chain damage. Moreover, ischemia–reperfusion injury (IRI) develops and occurs in a number of organs, including the liver, heart, and brain, as a result of ROS-induced vascular endothelial dysfunction. Additionally, ROS can set off inflammatory pathways that result in the no-reflow phenomenon, endothelial cell edema, and leukocyte aggregation. When ROS overproduction exceeds the cell’s ability to clear them, oxidative stress ensues. An uncontrolled ROS burst damages the membrane and proteins, indirectly triggering the opening of the mitochondrial permeability transition pore (mPTP) and promoting activation of the apoptosis pathways [12–15].

### Intracellular calcium overload

Myocyte relaxation and contraction depend on calcium homeostasis. Hypoxia in cardiomyocytes leads to increased Na+ levels, decreased pH, and excess calcium. Cellular calcium overload via reverse mode Na+/Ca2+ exchange following sodium overload through the Na+/H+-exchanger, oscillatory release and reuptake of Ca2+ in the sarcoplasmic reticulum, lead to uncoordinated and excessive myofibrillar contractions, degradation of the cytoskeleton and sarcolemma by calpains, and excessive generation of reactive oxygen species collectively contribute to cell death [12, 16, 17]. Altered myofibrillar contractile proteins can lead to decreased Ca2+ sensitivity and impaired contraction even though cytosolic Ca2+ is increased. An oxidative burst causes significant increases in Ca2+ influx and ROS generation which mediate lethal myocardial reperfusion damage. Maintaining calcium homeostasis is therefore essential for myocardial cell growth [6, 12, 18, 19].

### Mitochondrial dysfunction

Cardiomyocytes have a high mitochondrial density because they require a lot of energy [20]. A crucial mediator in cellular metabolism, mitochondrial calcium initiates the synthesis of adenosine triphosphate (ATP). Nevertheless, cytoplasmic calcium overflow leads to mitochondrial calcium overload during ischemia–reperfusion which results in mitochondrial malfunction. This leads to apoptosis and reduced ATP production. The mPTP reopens during reperfusion after being closed during ischemia. Oxidative stress, abrupt intracellular pH correction, and an excess of calcium and phosphate in the mitochondria are the reasons for mPTP opening. IRI causes ROS levels to rise quickly, which leads to an excess of cytoplasmic and mitochondrial Ca2+. As a result, the mitochondrial membrane potential opens and the mPTP depolarizes. Cellular expansion, ATP depletion, and cytochrome c (CytC) release follow, all of which set off apoptotic pathways [12, 21–23]. In addition, mitophagy—the autophagic clearance of damaged mitochondria—normally serves as a quality control mechanism to limit I/R injury. Specifically, the PINK1/Parkin pathway labels dysfunctional mitochondria for degradation, preventing excess ROS production and cell death [24, 25]. In fact, pharmacological activation of PINK1/Parkin-mediated mitophagy is cardioprotective [24], whereas deletion of PINK1 exacerbates cardiac I/R damage [25].

However, if reperfusion injury is severe, the burden of damaged mitochondria can overwhelm mitophagy, and excessive mitophagy may paradoxically promote cell death. For example, studies show that too much PINK1/Parkin activity during reperfusion can drive apoptosis, suggesting that an optimal balance of mitophagy is required to protect heart cells [24].

### Regulated modes of cell death

Apoptosis is a regulated process of cell death that results in the creation of apoptotic bodies, cytoplasmic and nuclear condensation, and cell contraction. Both intrinsic (such as hypoxia, hyperthermia, and low growth factors) and extrinsic (like transmembrane death receptors) mechanisms may trigger it. Ischemia and reperfusion during MIRI increase ROS levels, cause cellular damage, and impair circulatory function, which in turn triggers cardiomyocyte death. The Fas pathway plays a key role in mediating the cardiomyocyte death brought on by MIRI [12].

Both necroptosis and pyroptosis are forms of cell death caused by elevated interleukin-1 beta (IL-1β) levels, damage-associated molecular patterns, and tumor necrosis factor (TNF) receptor activation. Pyroptosis induces gasdermin-dependent pores to form, whereas necroptosis involves specific serine or threonine protein kinases, necrosome formation, and pore formation in the sarcolemma. Ferroptosis is a distinct type of iron-dependent, non-apoptosis-regulated cell death triggered by the breakdown of glutathione-dependent antioxidant defense systems and the buildup of lipid peroxides, both of which are caused by elevated ROS levels associated with Fe2+ [12, 26].

Importantly, these death pathways interconnect. For example, necroptotic signaling can activate pyroptosis—MLKL (a key necroptosis protein) forms pores that drive NLRP3 inflammasome activation and thus pyroptosis [27]. Ferroptosis can also be brought on by an excess of ROS generated during necroptosis or pyroptosis [28]. In turn, autophagy generally suppresses apoptosis by clearing damaged organelles that would otherwise promote cell death. These cross-activations imply that inhibiting one pathway may influence others. Thus, therapeutic strategies targeting one form of RCD in MIRI must consider effects on related pathways [27, 28].

Implications for therapy: Recognizing this cross-talk means therapies could be designed to modulate several pathways simultaneously. For example, antioxidants that lower ROS may lessen ferroptosis and pyroptosis, and inhibitors of necroptosis may also stop secondary pyroptotic harm. Overall, understanding these interactions is crucial for developing targeted interventions to protect the heart from reperfusion injury [27, 28].

## Clinical manifestations of reperfusion injury in STEMI

**Fig. 1:**
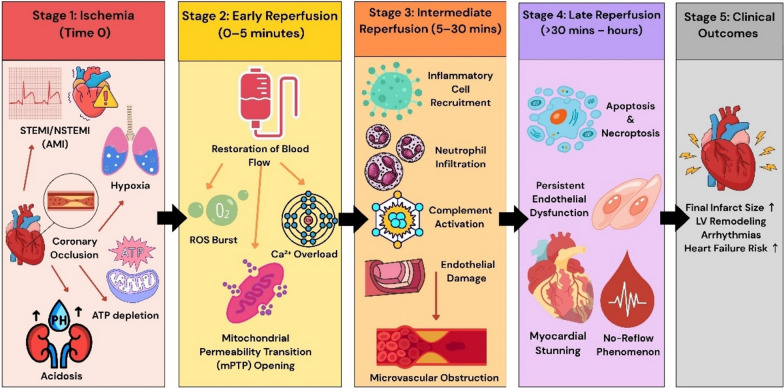
Timeline and mechanisms of reperfusion injury following therapy for STEMI. This schematic diagram traces five progressive stages from the moment of coronary occlusion. Stage 1 (Ischemia, Time 0)—coronary occlusion causes acute myocardial ischemia with hypoxia, ATP depletion, metabolic acidosis, and electrical instability that set the substrate for injury. Stage 2 (Early reperfusion, 0–5 min)—restoration of blood flow (for example with primary percutaneous coronary intervention) triggers an immediate burst of reactive oxygen species (ROS), rapid Ca^2+^ influx/overload and opening of the mitochondrial permeability transition pore (mPTP), promoting mitochondrial dysfunction and cell injury. Stage 3 (Intermediate reperfusion, 5–30 min)—innate inflammatory responses are recruited (neutrophil infiltration, complement activation) and endothelial damage contribute to microvascular obstruction. Stage 4 (Late reperfusion, >30 min–hours)—progressive cell death pathways (apoptosis, necroptosis), persistent endothelial dysfunction, myocardial stunning, and the no-reflow phenomenon amplify tissue loss. Stage 5 (Clinical outcomes)—these pathophysiological events translate into larger final infarct size, adverse left ventricular (LV) remodeling, increased arrhythmia risk, and higher likelihood of heart failure or recurrent ischemic events. Arrows in the figure indicate temporal progression and causal links between processes. STEMI—ST-segment elevation myocardial infarction; NSTEMI—Non-ST-segment elevation myocardial infarction; AMI—acute myocardial infarction; ROS—reactive oxygen species; Ca^2+^—calcium ion; mPTP—mitochondrial permeability transition pore; ATP—adenosine triphosphate; LV—left ventricle/left ventricular

Injury from reperfusion in STEMI manifests in four main forms namely myocardial stunning, no-reflow phenomenon, reperfusion arrhythmia, and lethal reperfusion injury [29, 30]. Figure 1 illustrates the progression from ischemia to myocardial reperfusion, highlighting key stages where reperfusion injury mechanisms are activated.

### Myocardial stunning

Following blocked coronary artery reperfusion, myocardial stunning is a persistent mechanical heart failure that is reversible and mostly related to the recovery of systolic and diastolic function [30]. Approximately 50% of STEMI patients treated with reperfusion exhibit myocardial stunning [31].

The processes include troponin-I degradation, calcium overload, excitation–contraction uncoupling, and a decrease in the myofilaments'calcium responsiveness [32]. The stunned myocardium uses more oxygen, which lowers its mechanical efficiency. The quick intracellular pH restoration that occurs during reperfusion is partially to blame for this [30]. Imaging methods used to identify myocardial stunning include gated Tc-99m single-photon emission computed tomography, myocardial contrast echocardiography, and dobutamine echocardiography. Myocardial temperature, the extent and duration of blood flow deprivation, and the heart's loading conditions all affect how severe stunning is [32].

### No-reflow phenomenon

After undergoing reperfusion therapy, individuals with STEMI frequently experience coronary no-reflow (CNR) [16]. Incidence estimates vary: One large registry found angiographic no-reflow in only ~1–3% of STEMI PCI cases [33], whereas Khalfallah et al. [34] reported a 13.9% no-reflow rate in 545 STEMI cases. Because definitions and populations vary, reported rates range widely—roughly 2–44% across studies [35]. This is a dynamic phenomenon that might change within a few hours and possibly last for days or weeks. The primary pathophysiological cause of CNR is microvascular obstruction (MVO), which is brought on by ischemia and presence of thrombotic material further downstream in the affected vessel. ROS are created as a result of intracellular alterations during MIRI and they harm lipids, proteins, and DNA in cells. Microvascular hypoperfusion is worsened, and infarct size is increased by endothelial degradation. Microvascular blockage is caused by neutrophil accumulation brought on by myocyte enlargement and tissue edema [16]. According to Akpek Met et al., patients without reflow had higher rates of cardiogenic shock, malignant arrhythmias, cardiac rupture, cardiac mortality, and major adverse cardiovascular/cerebrovascular events (MACCE) [36].

### Reperfusion arrhythmias

The first 48 h after MI is extremely important since these patients are prone to reperfusion arrhythmias such as atrial fibrillation, sinus bradycardia, sinus tachycardia as well as non-sustained ventricular tachycardia and accelerated idioventricular rhythm [30]. It affects 4–5% of STEMI patients who have had PPCI making it a quite common disease [37]. More recent continuous-monitoring data indicate reperfusion ventricular arrhythmias occur in a much higher proportion (~48.8%) of reperfused STEMI patients [38]. In the Osaka Acute Coronary Insufficiency Study, a significantly greater incidence was noted: within 12 h of the beginning of symptoms 23% of patients who got PPCI experienced reperfusion-related ventricular arrhythmia [37]. Factors like acidosis, α-adrenergic stimulation, and angiotensin II release can increase the ischemic myocardium's susceptibility to reperfusion arrhythmias and increased autonomic stimulation of Purkinje fibers near ischemic regions may accelerate idioventricular rhythm thus posing a challenge to cardiologists [30].

### Lethal reperfusion injury

Lethal reperfusion damage is the paradoxical stimulation of myocyte death following resuscitation of the ischemic myocardium [29]. The mitochondrial inner membrane permeability during reperfusion determines the fate of cells; low permeability permits cell recovery, moderate permeability causes programmed cell death, and high permeability causes necrosis. The main cause of cardiomyocyte mortality during MIRI is necrosis, a controlled process [29].

Lethal reperfusion injury is largely caused by mPTP, and modifying it is a possible therapeutic target to stop reperfusion injury in the future. According to experimental models, between 50 and 75 percent of the final infarct size may be due to lethal reperfusion injury [30]. Prevention of deadly reperfusion damage is especially crucial since it increases the extent of the myocardial infarct and has a direct impact on the prognosis for patients with STEMI. However, no strategy has been effective in preventing this harm yet [29].

### Intramyocardial hemorrhage

In addition to the forms mentioned above, cardiac magnetic resonance imaging has been used to document intramyocardial hemorrhage (IMH) which follows STEMI [39]. IMH can result from blocked areas that are accompanied by microvascular injury and may expand the hypoxic zone after revascularization by exacerbating microvascular dysfunction through vasospasm and external compression. Furthermore, cardiomyocytes may be exposed to heme, a cytotoxic iron-binding component of hemoglobin, as a result of hemolysis of red blood cells in the extracellular environment. Consequently, both hemodynamic compromise and cytotoxic effects of heme may contribute to infarct expansion in the setting of IMH [40]. MRI studies of reperfused STEMI patients consistently report IMH in a substantial minority. A recent meta-analysis (*n* = 2824) found IMH in ≈39% of patients. IMH is linked to a bigger infarct size, a higher left ventricular end-diastolic volume (LVEDV), and a lower left ventricular ejection fraction (LVEF) following STEMI. It is also an accurate marker of MACCE [39].

### Hemodynamic changes

Patients with PPCI may exhibit a range of clinical characteristics and coronary angiography findings due to occlusive infarct-related artery or balloon predilation. While some patients have stable vital signs, others suffer blood pressure and heart rate drops; malignant arrhythmia, no-reflow, and cardiogenic shock can also occur. The viable myocardium may thus be reflected in the clinical manifestations during cardiac reperfusion; more pronounced symptoms may indicate a more viable myocardium [41]. A study by Li et al. found that 34 patients out of 80 with STEMI undergoing primary PCI exhibited clinical features of ischemia–reperfusion injury, including severe bradycardia, hypotension, refractory ventricular arrhythmias, and no-reflow or slow-flow on angiography [42]. The prognosis can be improved or reperfusion damage symptoms can be avoided with interventions [41].

Optimizing results for STEMI patients undergoing revascularization requires early identification and treatment of these various reperfusion damage symptoms.

## Imaging, biomarkers, and outcomes of reperfusion injury in STEMI

Infarct size is significantly decreased in STEMI when coronary blood flow is promptly restored by PPCI. However, the reperfusion process can affect the myocardium itself. Reperfusion injury is common with cardiac MRI studies finding MVO in ~40–50% of reperfused STEMI patients [30, 43] and IMH in ≈40% [39]. This injury is clinically significant because: With modern percutaneous coronary intervention (PCI), STEMI mortality is ~7% at 1 year and 22% of the patients develop heart failure [44]. Therefore, minimizing reperfusion injury could improve infarct salvage and long-term outcomes.

### Diagnostic imaging

#### Cardiac magnetic resonance (CMR) imaging

Cardiac MRI is the most precise imaging technique for identifying reperfusion injury. Late gadolinium enhancement sequences delineate infarct size and areas of microvascular obstruction (hypo enhancement). Myocardial swelling and IMH are identified by T2-weighted imaging. [39, 45]. In a recent study, persistent MVO on MRI (seen in ≈10% of cases) was associated with larger infarcts, lower LVEF, and more frequent hemorrhage [45]. Thus, MRI can quantify infarct size, MVO, and IMH as predictors of remodeling. However, MRI is costly and less accessible, often limited to specialized centers and developed countries [46]. While MRI provides high spatial resolution and detailed tissue characterization [47], it requires breath-holds (has moderate temporal resolution) and cannot be used in unstable patients or those with certain implants [48].

#### Echocardiography

Standard echo assesses global and regional function after STEMI (e.g., LVEF, wall motion). While sophisticated methods such as myocardial contrast echo or speckle-tracking strain [49] can identify subtle perfusion abnormalities, MRI is still more sensitive for MVO/IMH [50]. Ventricular remodeling can be serially assessed using echocardiography**.** When compared to MRI, echocardiography is inexpensive and less invasive [51]. It offers real-time imaging with excellent temporal resolution, is portable, and can be done at the bedside [52]. However, it is operator dependent [52], may have poor penetration due to air trapping in patients with lung diseases like COPD [53] or obesity, and provides limited tissue characterization [54].

#### Coronary angiography

Coronary angiography (invasive X-ray angio) is the gold standard for defining coronary anatomy and allows immediate interventions [55]. The thrombolysis in myocardial infarction (TIMI) coronary grade flow is a useful and thorough coronary reperfusion grading method on an angiography [56]. At the time of PCI, epicardial flow is graded by TIMI risk score and myocardial perfusion by myocardial blush grade. No-reflow (TIMI ≤ 2 or poor blush) indicates reperfusion injury at the microvascular level. New angiographic indices quantify microvascular resistance: One study found an angiography-derived microcirculatory resistance (AMR) cutoff > 2.7 mmHg s/cm predicted MRI-detected MVO (area under the curve [AUC] = 0.821). Elevated AMR identifies patients at high risk for STEMI, including those progressing to heart failure (hazard ratio HR ≈ 2.15, 95% CI 1.43–3.22; *P* < 0.001) [57]. Thus angiographic measures correlate with MVO and prognosis.

Despite being the gold standard, coronary angiography is costly and fraught with dangers, such as problems at the vascular access site and nephropathy brought on by contrast material [58]. As a low-risk, non-invasive substitute, coronary-computed tomography angiography (CCTA) is gaining traction. Using a contrast agent, a radiographic evaluation of the epicardial coronary arteries is part of this diagnostic procedure. Even the farthest coronary artery segments may now be seen thanks to CCTA's improved spatial and temporal resolution [59]. To summarize, each modality has trade-offs in cost, access, and resolution, which are depicted in Table 1.

**Table 1:** Comparison of imaging modalities for detection of reperfusion injury in STEMI

Features	Imaging modalities			
	Cardiac magnetic resonance (CMR) imaging	Echocardiography	Coronary angiography	Coronary CT angiography (CCTA)
Strengths	Very high spatial resolution and tissue characterization (infarct size, MVO, IMH); quantitative tissue mapping available	Portable, bedside-capable, no radiation; low cost; real-time wall motion and functional assessment; advanced techniques (speckle-tracking) improve sensitivity	Gold-standard for coronary anatomy; immediate PCI capability; direct assessment of epicardial flow (TIMI) and myocardial blush during procedure	Non-invasive evaluation of epicardial coronary arteries; improving spatial and temporal resolution allows visualization of distal segments; useful low-risk alternative for anatomical assessment
Limitations	High cost; limited availability; not suitable for unstable patients or those with certain implants; longer scan times	Operator-dependent; moderate spatial resolution; less sensitive for microvascular obstruction (MVO) and intramyocardial hemorrhage (IMH) than CMR	Invasive; radiation and iodinated contrast exposure; cannot directly image myocardial tissue (MVO inferred indirectly); requires cath lab	Radiation and contrast exposure; less useful acutely in unstable patients; limited ability to assess microvascular injury (MVO/IMH) and no immediate therapeutic capability
Cost	High	Low	Moderate–High	Moderate
Accessibility	Limited (tertiary/specialized centers)	Very high (widely available in most hospitals/clinics)	High (PCI-capable centers/cath labs)	Increasing availability (CT-capable centers); more accessible than CMR in many settings
Temporal Resolution	Moderate (cine imaging; seconds per frame; multiminute protocols)	Very high (real-time, milliseconds/frame)	Very high (real-time fluoroscopy; milliseconds)	Improving-modern scanners approach high temporal resolution (milliseconds); multicycle and dual-source techniques enhance temporal resolution
References	[39, 45–48]	[49, 50, 52]	[55–57]	[59]

Table shows the comparison of strengths, limitations, cost, accessibility, temporal resolution, and representative references for echocardiography, CMR, invasive coronary angiography, and CCTA when used to evaluate reperfusion injury after STEMI. MVO = microvascular obstruction; IMH = intramyocardial hemorrhage; PCI = percutaneous coronary intervention; TIMI = thrombolysis in myocardial infarction; CMR = cardiac magnetic resonance; CCTA = coronary CT angiography

### Biomarkers

#### Cardiac enzymes [creatine kinase-MB (CK-MB), troponin]

Peak CK-MB (≥300 U/L in cohort studies as a cut-point associated with large infarct size and worse outcomes) and high-sensitivity troponin reflect infarct necrosis. Significant pre-PCI markers of subsequent microvascular damage in MVO prediction models were increased troponin T and CK-MB. In practice, higher peak troponin/CK-MB levels generally indicate larger infarcts and have been linked to worse left ventricular (LV) function and outcomes [43].

#### Serum soluble growth stimulation expressed gene 2 protein (sST2)

Cardiomyocytes and fibroblasts release the soluble growth stimulator gene 2 protein (sST2) in response to cardiovascular and/or mechanical stress [60]. Recent studies have defined prognostic thresholds for sST2 in STEMI [61]. A poor prognosis for myocardial infarction is linked to a greater sST2 level [60]. For example, sST2 levels > 35 ng/mL have been Linked to significantly higher 30-day mortality and heart failure risk [61]. Other cohorts suggest cutoffs around 48–69 ng/mL for predicting MACE [62, 63]. In a 2025 cohort study, STEMI patients with post-PCI reperfusion injury had significantly higher sST2; an sST2 > 68.98 ng/mL was an independent predictor of mortality and major adverse cardiac and cerebrovascular events (MACCE) [63]. According to Aleksova et al. [61], an intermediate sST2 threshold range of approximately 35–70 ng/mL is often cited for risk stratification, identifying patients at increased risk for adverse remodeling and poor outcomes, thus aiding clinical decision-making postreperfusion.

Apart from SST2, Galectin-3 (Gal-3) is a potentially useful prognostic biomarker that is implicated in inflammation, fibrosis, and remodeling of the heart in response to myocardial injury [64]. The β-galactoside-binding lectin Galectin-3 (Gal-3) is mostly released by activated macrophages [65]. It has been demonstrated that both soluble ST2 (sST2) and galectin-3 rise after myocardial infarction [66]. However, sST2 retains independent prognostic value even after adjusting for Galectin-3 [67]. In practice, sST2 interferes with IL-33/ST2L signaling by acting as a decoy receptor [66], whereas Galectin-3 is a marker of fibrosis/macrophage activity [64]. Both can be elevated in post-AMI remodeling. In one AMI cohort (aortic-root sampling during angiography; *n* = 59), Galectin-3 ≥ 10.86 ng/mL predicted 6-month MACE or death (AUC 0.858, 95% CI 0.744–0.973; sensitivity 80%, specificity 87%) [64].

In addition, increased levels of hs-CRP indicate heightened inflammatory states, making it a well-established indicator of systemic inflammation. In both infectious and non-infectious settings, hs-CRP accurately represents inflammatory burden and stratifies cardiovascular risk [68]. For risk prediction in STEMI, studies indicate that sST2 frequently performs better than hs-CRP. For example, sST2 had a higher AUC for 30-day MACE than hs-CRP [62].

#### Other biomarkers

B-type natriuretic peptide (BNP) and N-terminal pro-B-type natriuretic peptide (NT-proBNP) rise in large infarcts or when heart failure develops [69]. For example, in non-acute settings, the upper reference Limit for NT-proBNP is 125 pg/mL, whereas in acute presentations the thresholds increase to 300 pg/mL [70]. This implies that the limits exceed significant LV stress post-MI.

Acute MI triggers a systemic inflammatory response [71]. CRP is an acute-phase protein induced by IL-6 and a well-known risk marker—levels > 3 mg/L (hs-CRP) denote high cardiovascular risk [72]. Elevated IL-6 and TNF-α (upstream cytokines) post-STEMI are also independently associated with worse outcomes [73]. It has been reported that an increased risk of myocardial infarction, stroke, peripheral arterial disease, heart failure, and significant adverse cardiovascular events is linked to elevated levels of IL-6 [74]. Inflammasome-derived cytokines including IL-1β and IL-18 are increased after myocardial infarction, which leads to myocardial damage and subsequent contractile dysfunction [75]. Therefore, biomarkers correspond with infarct size and reperfusion injury. However, sST2 appears to be a stronger independent prognostic marker in recent cohorts [63]. Key biomarkers involved in the detection and prognosis of reperfusion injury are summarized in Table 2.

**Table 2:** Key biomarkers for diagnosis and prognosis of reperfusion injury in STEMI

Biomarker	Function/role	Study type/population	Timing of measurement	Cutoff	Diagnostic/prognostic notes	References
CK-MB	Marker of myocardial necrosis	Cohort/STEMI (post-PCI)	Peak after PCI	≥300 U/L	Peak CK-MB predicts infarct size and is linked to poorer LV function and outcomes	[43]
hs-Troponin T (hs-cTnT)	High-sensitivity marker of myocardial injury	Cohort/STEMI (pre- and post-PCI)	Admission/peak/24 h	Exact value not specified	Elevated pre-PCI hs-TnT predicts MVO and correlates with infarct size and LV dysfunction	[43]
sST2	Soluble IL-33/ST2 axis—cardiac stress/inflammation	Cohort studies; STEMI (post-PCI)	Admission/24 h	>35 ng/mL (risk); ~48–70 ng/mL (MACE in some cohorts); >68.98 ng/mL (mortality/MACCE in a cohort)	sST2 appears to outperform hs-CRP for short-term MACE in several cohorts; higher sST2 independently predicts reperfusion injury, mortality, and MACCE	[60–63]
NT-proBNP	Marker of myocardial wall stress/heart failure	Cohort/STEMI	24–48 h post-PCI (acute)	Acute: 300 pg/mL (rule-out threshold); Non-acute upper Limit: 125 pg/mL	Elevated post-STEMI (large infarcts) predicts adverse remodeling and HF risk	[69, 70]
Inflammatory biomarkers (specific: hs-CRP, IL-6, TNF-α, IL-1β, IL-18)	Circulating mediators of systemic inflammation after MI	Cohort/STEMI	Peak/24–48 h	hs-CRP > 3 mg/L; IL-6 and other cytokines: no universal cutoff (example IL-6 ≥ 9.5 pg/mL reported in cohorts)	hs-CRP indicates inflammatory burden and stratifies CV risk; IL-6/TNF-α/IL-1β/IL-18 linked to worse outcomes and remodeling	[71–75]; hs-CRP specifically [72]
Galectin-3 (Gal-3)	Marker of macrophage activation, fibrosis and remodeling	Prospective cohort; first-AMI patients undergoing angiography; *n* = 59 (aortic-root sampling)	Within 24 h of chest-pain onset/at angiography	≥10.86 ng/mL (ROC-derived, cohort-specific)	Predicted 6-month MACE/death in the cohort (AUC 0.858; 95% CI 0.744–0.973; sensitivity 80%, specificity 87%). Note: cohort-derived and requires external validation (small sample)	[64]

Cutoffs are assay- and cohort-dependent; values shown are guideline-recommended where available or cohort-derived from cited studies. Inflammatory cytokine levels (e.g., IL-6, IL-1β, TNF-α) vary by assay and timing—report assay, sample type (serum/plasma), and time from symptom onset and/or PCI. Troponin sampling: admission (0 h), serial at 3–6 h, and peak/24 h (peak or 24-h value preferred for infarct size/prognostic correlations). Units are shown where applicable (e.g., sST2 in ng/mL). MACE = composite of cardiovascular death, recurrent MI, stroke, heart failure hospitalization, or urgent revascularization. hs-cTnT—high-sensitivity cardiac troponin T; NT-proBNP—N-terminal pro-B-type natriuretic peptide; hs-CRP—high-sensitivity C-reactive protein; sST2—soluble ST2; Gal-3—Galectin-3; CK-MB—creatine kinase-MB; MVO—microvascular obstruction; MACCE—major adverse cardiac and cerebrovascular events; IMH—intramyocardial hemorrhage; LVEF—left ventricular ejection fraction; LOD—limit of detection; URL—upper reference limit; MACE—major adverse cardiovascular events; PCI—percutaneous coronary intervention; AUC—area under the ROC curve; CI—confidence interval; TNF-α—tumor necrosis factor alpha; IL—interleukin (e.g., IL-6, IL-1β, IL-18); HF—heart failure; ROC—receiver operating characteristic

### Impact on clinical outcomes

Reperfusion injury strongly worsens outcomes. In the context of reperfused STEMI, “Major Adverse Cardiovascular Events (MACE)” typically means a composite of cardiovascular death, recurrent MI, stroke, heart failure, or urgent revascularization [73]. It is a frequent outcome of cardioprotection and reperfusion damage experiments. Large studies have quantified how reperfusion injury markers relate to MACE. In a STEMI cohort, in-hospital MACE was roughly twice as likely to occur in patients with reperfusion-related ventricular arrhythmias (HR = 2.173; 95% CI 1.03–4.667; *p* = 0.021) [76].

A meta-analysis of 18 studies (2824 STEMI patients) demonstrates that IMH, present in approximately 39% of cases, was associated with a higher risk of major adverse cardiovascular events (MACE) (OR 2.63; 95% CI 1.79–3.86; *P* < 0.00001) [39]. The same meta-analysis also reported associations between IMH and TIMI grade < 3 after PCI (OR 1.75; 95% CI 1.14–2.68; *P* = 0.05) and between IMH and the use of glycoprotein IIb/IIIa inhibitors (OR 2.34; 95% CI 1.42–3.85; *P* = 0.0008). IMH was further associated with larger infarct size [standardized mean difference (SMD) 2.19; 95% CI 1.53–2.86; *P* < 0.00001], greater LV end-diastolic volume (SMD 0.70; 95% CI 0.41–0.99; *P* < 0.00001), and lower LVEF (SMD −0.89; 95% CI −1.15 to −0.63; *P* = 0.01). Among the factors that predicted IMH were smoking, male sex, and left anterior descending (LAD) infarct [39].

Poor outcomes are also predicted by MRI findings of microvascular obstruction (MVO); according to one study, MVO was an independent predictor of 1-year MACE (HR 3.94; 95% CI 1.00–10.25; *p* = 0.049) [77]. Clinically, these imaging findings translate to patient events. For example, in a recent cohort study, the angiographic no-reflow phenomenon was seen in 4.2% of STEMI patients and it was associated with a 1-year combined event rate of 23.2% versus 12.2% with the normal flow. Coronary no-reflow (TIMI ≤ 2) occurs in a minority of modern STEMIs but carries risk. In short, patients with evident microvascular reperfusion injury have significantly higher rates of death, recurrent MI, and heart failure than those without [78]. In summary, these findings indicate that patients with STEMI are at significantly increased risk for mortality, heart failure, or another ischemic event when reperfusion injury is substantial, as in the case of a big infarct, persistent MVO or IMH or arrhythmias [39, 44]. It also underscores the importance of identifying high-risk patients (via MRI, angiography, and biomarkers like troponin and sST2) and developing therapies to mitigate reperfusion injury.

## Therapeutic strategies for reperfusion injury in STEMI

Cardioprotective measures that fall into the mechanical and pharmacological categories lessen the paradoxical tissue damage brought on by reperfusion injury after STEMI.

### Mechanical strategies

Several mechanical methods have been studied to enhance myocardial recovery in STEMI patients. Early proof-of-concept studies suggest that remote ischemic conditioning (RIC), which temporarily blocks blood flow to a limb before restoring blood flow to the heart, may help reduce the severity of a cardiac arrest. Because it can be administered before, during, or after coronary reperfusion and acts throughout the body, RIC is a desirable non-invasive treatment option [26]. Three cycles of remote ischemic conditioning applied to the leg in addition to standard of care improved the outcome of patients with STEMI when compared to standard of care alone, according to a 2018 RIC-STEMI clinical trial (Gaspar et al. [79]; *n* = 258) that included cardiac-related mortality and heart failure hospitalization (HR = 0.35, 95% CI 0.15–0.78) as primary end points [79]. However, in the larger CONDI-2/ERIC-PPCI trial (*n* = 5401) RIC applied to an arm immediately before primary PCI did not reduce infarct size, as assessed by high-sensitivity troponin T level, or improve outcomes in patients with STEMI (event rates 8.6% vs. 9.4%; HR 1.10, 95% CI 0.91–1.32; *p* = 0.32) [80]. Recent reviews recommend that RIC should be targeted to patients at high risk of extensive tissue injury (for example those with delayed hospital admission, high Killip class, cardiogenic shock, or cardiac arrest), and that broad application in lower-risk, contemporary STEMI cohorts may dilute any treatment effect [81]. Thus, the neutral findings of large multicenter trials could reflect population heterogeneity and low-event rates rather than lack of biological efficacy alone.

Ischemic postconditioning (IPoC) has also been studied. Heusch notes that ischemic postconditioning, in which brief cycles of reocclusion–reperfusion are delivered after opening the artery, can be used in patients with acute myocardial infarction and has shown promise in proof-of-concept clinical trials [26]. Moreover, a combined mechanical strategy by applying IPoC together with arm RIC just before reperfusion appears beneficial as this approach improved myocardial salvage by cardiac MRI in the randomized LIPSIA CONDITIONING trial (*n* = 696, difference significant; *p* ≈ 0.02) [82].

CARIOCA (NCT03155022) is a randomized trial that compares combined RIC with intracoronary/ischemic postconditioning versus standard primary PCI. It is listed on ClinicalTrials.gov with a planned enrollment of ≈750 and status “active, not recruiting”; no trial results were posted on the registry yet [83].

Researchers examined whether cooling the body with endovascular methods and cold saline could reduce Heart damage following a Heart attack in the CHILL-MI experiment, which involved 120 patients. Prior to reperfusion, the core temperature rose to 34.7 °C, and cooling was safe. The median infarct size in relation to the area at risk was 40.5% with cooling against 46.6% in controls (a 13% relative reduction, *p* = 0.15), but overall, it did not significantly lower infarct size. Mean symptom onset to randomization 129 ± 56 min (hypothermia) versus 132 ± 64 min (controls) door-to-balloon time was delayed by roughly nine minutes Due to cooling. There was no difference in mortality at about 45 days; however, heart failure was less common in the cooled group (3% vs. 14%, *p* < 0.05). [84]. As Heusch reports, clinical trials on therapeutic hypothermia in small cohorts of patients with STEMI have so far not demonstrated a significant reduction in infarct size [26]. This disappointing result is attributed to the practical difficulties of achieving rapid, effective myocardial cooling during acute MI.

Table 3 summarizes key human clinical trials and ongoing studies of mechanical strategies to prevent myocardial reperfusion injury in STEMI.

**Table 3:** Mechanical cardioprotective interventions in STEMI: key human trials

Trial (year)/study type	Intervention	Comparator	Sample size (*N*)	Major numerical outcomes	Status	Design limitations/likely reasons for neutral/failure	Potential alternatives	References
RIC-STEMI (Gaspar et al. 2018)—RCT	3 cycles remote ischemic conditioning applied to the leg + standard of care	Standard of care alone	258	Primary composite (cardiac-related mortality and HF hospitalization): HR = 0.35 (95% CI 0.15–0.78)	Positive (single-center positive RCT)	Single-center RCT (proof-of-concept size limited generalizability)	Test in larger/enriched cohorts and/or combined strategies	[79]
CONDI-2/ERIC-PPCI (Hausenloy et al. 2019)—multicenter RCT	Upper-limb RIC immediately before primary PCI (arm cuff cycles)	Standard care	5401	Event rates 8.6% versus 9.4%; HR 1.10 (95% CI 0.91–1.32); *p* = 0.32 (no reduction in infarct size/no outcome benefit)	Neutral (large RCT)	Large, contemporary low-event-rate population; heterogeneity of RIC algorithms may dilute effect	Enrich for high-risk patients (delayed presenters, high Killip, shock/arrest); test multimodal strategies	[80]
LIPSIA CONDITIONING (Eitel et al. 2015)—RCT	Combined IPoC + arm RIC applied before reperfusion	Control/other strategies (per trial design)	696	Improved myocardial salvage by CMR (difference significant; *p* ≈ 0.02)	Positive (improved myocardial salvage)	Intermediate-size trial; may reflect selection/protocol differences versus larger trials	Investigate combined conditioning	[82]
CARIOCA (ClinicalTrials.gov NCT03155022)—randomized	Combined remote ischemic perconditioning + intracoronary/ischemic postconditioning	Standard primary PCI	Planned ~ 750	Not yet published	Active, not recruiting	No results yet	None yet	[83]
CHILL-MI (Erlinge et al. 2014)—RCT (therapeutic hypothermia)	Rapid endovascular catheter core cooling + cold saline as adjunct to PCI	Standard care (control)	120	Symptom-to-randomization 129 ± 56 min versus 132 ± 64 min; core temp 34.7 °C before reperfusion; door-to-balloon + 9 min; median IS/MaR 40.5% (IQR 29.3–57.8) versus 46.6% (IQR 37.8–63.4) (relative reduction 13%; *p* = 0.15); HF incidence ~45 days 3% versus 14% (*p* < 0.05); no difference in mortality	Neutral overall for infarct size; exploratory HF/early-anterior signals noted	Small sample; difficulty achieving rapid myocardial cooling; possible delay to reperfusion	Larger/enriched trials or improved cooling delivery methods; consider targeting early-anterior MI subgroup	[84]

Summary of key human trials of mechanical cardioprotective strategies (e.g., remote ischemic conditioning [RIC], ischemic postconditioning [IPoC], therapeutic hypothermia). Columns report study design, detailed intervention and comparator, sample size (N), principal numerical outcomes (including hazard ratios [HR] with 95% confidence intervals [CI], p-values and interquartile ranges [IQR]), trial status, design limitations, and the most plausible explanations for neutral or negative outcomes, along with potential alternatives and references. Only human data are included. RIC = remote ischemic conditioning; IPoC = ischemic postconditioning; RCT = Randomized Control Trial; PCI = percutaneous coronary intervention; CMR = cardiac magnetic resonance; IS = infarct size; MaR = myocardium at risk; HF = heart failure

### Pharmacological strategies

Pharmacological strategies involve medications designed to mitigate tissue damage driven by reperfusion-associated pathophysiology. According to Kakavand et al. [85], since MIRI involves oxidative stress, calcium overload, microvascular obstruction, and inflammation, drugs for its prevention can be classified by mechanism into antioxidants, metabolic modulators, rheological (microcirculation) agents, anti‐inflammatories, and multitarget drugs.

#### Drugs that reduce oxidative stress

N-acetylcysteine (NAC) is a glutathione precursor and free radical scavenger. In recent randomized trials of STEMI patients, high-dose IV NAC (often given with nitrates) significantly reduced markers of myocardial injury and improved reperfusion. For example, NACIAM (N-acetylcysteine + nitrate) a double-blind RCT found NAC doubled the myocardial salvage index and reduced infarct size on MRI (median 11.0% vs. 16.5% of LV mass in placebo; *P* = 0.02) [86]. Another trial from 2018 showed NAC accelerated troponin decline and increased TIMI grade-3 flow rates (*n* = 100, TIMI grade-3 flow: 94% (NAC) vs. 80% (placebo), *p* = 0.03; accelerated troponin decline) [87]. These studies suggest NAC attenuates reperfusion injury by preserving microvascular flow and limiting necrosis [86, 87].

Ascorbate, or vitamin C, is a potent antioxidant that has also been investigated in STEMI patients. Administering a high dosage of vitamin C intravenously before PCI decreased Blood indicators associated with inflammation and cardiac injury in a meta-analysis of multiple RCT with 1185 patients. The studies did not, however, consistently demonstrate benefits in patient outcomes or cardiac imaging, most likely due to the fact that heart damage following a heart attack occurs through a multiple routes other than oxidative stress [88]. In summary, antioxidant strategies have reduced oxidative biomarkers in humans, but none has definitively reduced infarct size or improved outcomes as monotherapy.

#### Drugs that affect cellular metabolism

SGLT2 inhibitors modulate metabolism by shifting cardiac substrate use. In the large DAPA-MI trial (2023), STEMI patients without diabetes were randomized to dapagliflozin versus placebo soon after MI. Dapagliflozin significantly improved a composite of cardiometabolic outcomes (e.g., diabetes onset, weight loss) compared to placebo, but did not reduce the combined endpoint of death or heart failure (HR ≈ 0.95; 95% CI 0.64–1.40) at 1 year [89]. Thus, SGLT2 inhibition showed metabolic benefits but no clear effect on immediate post-MI remodeling or function within 1 year.

Trimetazidine: This metabolic modulator shifts myocardial fuel from fatty acids to glucose. A recent meta-analysis (14 RCTs) found that pre-PCI trimetazidine significantly *reduced periprocedural troponin-I release* and improved ejection fraction compared to control [90]. In other words, trimetazidine given before reperfusion limits infarct size and preserves function.

#### Rheological agents targeting microvascular obstruction

Adenosine is an endogenous vasodilator that also inhibits platelet/neutrophil activation [91]. A 2024 meta-analysis of 21 RCTs (*n* = 2467 STEMI patients) found adjunctive adenosine markedly improved reperfusion parameters: It increased rates of ST-segment resolution (RR ≈ 1.30; *P* < 0.001) and significantly reduced no-reflow (RR ≈ 0.35; *P* < 0.001). Moreover, adenosine was associated with a lower risk of major adverse cardiac events (RR ≈ 0.67; *P* = 0.003) and reduced new heart failure (RR ≈ 0.66; *P* = 0.044) [92]. These human data indicate that intracoronary or IV adenosine during PCI enhances microvascular perfusion and can limit infarct extension.

Nicorandil, a hybrid ATP‐sensitive potassium channel opener with nitrate-like effects, has also shown benefit. In the randomized CHANGE trial (2022), STEMI patients received IV nicorandil (6 mg bolus + infusion) immediately before PCI. Nicorandil-treated patients had significantly smaller infarct sizes on cardiac MRI at 1 week (26.5 ± 17.1 g vs. 32.4 ± 19.3 g in placebo; *P* = 0.022). The nicorandil group experienced much less no-reflow (9.2% vs. 26.3%; *P* = 0.001) and more complete ST-resolution (90.8% vs. 78.0%; *P* = 0.006). Left ventricular ejection fraction was higher with nicorandil at 6 days (47.0% vs. 43.3%; *P* = 0.011) and 6 months (50.1% vs. 46.4%; *P* = 0.009) [93]. Such human trial results support that nicorandil improves microvascular flow and reduces infarct extent in STEMI.

Pentoxifylline is a methylxanthine with anti-inflammatory and antioxidant properties that increase RBC deformability. This was examined by Kakavand and colleagues in the recent PENTOS-PCI experiment, which involved 161 patients (2023). They explored whether administering pentoxifylline through an IV before primary PCI may enhance epicardial or microvascular flow. However, when compared to a placebo, the results showed no discernible difference. For instance, 71.3% of patients receiving pentoxifylline had the best amount of epicardial blood flow (TIMI-3) compared to 66.3% receiving a placebo (*p* = 0.40). The highest myocardial blush grade was Likewise comparable, at 87.5% versus 85.2% (*p* = 0.79), and there was no discernible improvement in other flow metrics [94]. Thus, despite theoretical rheologic benefits, pentoxifylline failed to reduce no-reflow or infarct size in that trial.

#### Anti-inflammatory agents

Colchicine, which inhibits neutrophil activation, was tested in acute STEMI (COVERT-MI trial) with an oral loading dose at reperfusion. This double-blind RCT found no reduction in infarct size by MRI at 5 days (*n* = 192, median ~26 g in colchicine vs. ~28 g in placebo; *P* = 0.87). There were also no differences in LV remodeling at 3 months, and colchicine caused more gastrointestinal side effects (34% vs. 11%, *p* = 0.0002) [95]. Thus, perireperfusion colchicine did not provide cardioprotection in this human trial.

Patients with STEMI were treated with tocilizumab (a medication that blocks the IL-6 receptor) in the ASSAIL-MI trial. The myocardial salvage index rose by 69.3% (compared to 63.6% for the placebo), an adjusted difference of 5.6 percentage points (*p* = 0.04), following a single intravenous dose administered prior to PCI. Tocilizumab recipients also had less microvascular obstruction on MRI. However, the final infarct size (percent of myocardium) was only numerically lower (7.2% vs. 9.1%; *P* = 0.08) and did not reach statistical significance [96]. These findings suggest IL-6 signaling contributes to reperfusion injury, since tocilizumab enhanced salvage in humans, although larger trials are needed to confirm outcome benefit.

In summary, while chronic anti-inflammatory therapy (e.g., canakinumab, colchicine in chronic CAD) can reduce events, acute anti-inflammatory therapy at reperfusion has generally yielded negative or equivocal results in humans to date [95, 96].

#### Agents with mixed mechanisms of action

Β-blockers (both Selective and non-selective β−1 antagonists) tend to reduce infarct size and also increase cardiac tolerance to reperfusion injury according to a systematic review in 2024 [97]. It was seen that metoprolol, a cardioselective β-blocker, is effective in reducing the progression of ischemic injury before reperfusion, achieves a smaller infarct size, induces QRS complex shortening on electrocardiogram (ECG), and increases LVEF when administered intravenously as soon as possible [98–101]. It may be administered during hospitalization and continued postdischarge [102]. This was also seen in the METOCARD-CNIC trial where intravenous metoprolol therapy (up to three 5-mg doses) given before reperfusion reduced infarct size, as assessed by creatine kinase release, in patients with STEMI.

Early MRI at one week in the 270-patient METOCARD-CNIC trial revealed that patients receiving IV metoprolol had an infarct that was roughly 20% smaller. Metoprolol was associated with a larger average LVEF at six months, according to MRI data (48.7% vs. 45.0%; adjusted effect 3.49%, 95% CI 0.44–6.55; *p* = 0.025). Additionally, the prevalence of severe LV dysfunction (LVEF ≤ 35%) was lower—11% as opposed to 27% in the control group (*p* = 0.006). Clinical composite trend at ~2 years: 10.8% vs. 18.3%; adjusted HR 0.55 (95% CI 0.26–1.04), *p* = 0.065 [103]. The later and lower-dose EARLY-BAMI trial, on the other hand, had no benefit, demonstrating the impact of both timing and dosage. *N* = 683 (metoprolol *n* = 336; placebo *n* = 347). CMR infarct size (%LV): 15.3 (metoprolol) versus 14.9 (placebo); *p* = 0.616 [104].

Cyclosporine A (CsA) prevents mPTP over-opening and has a role in cardioprotection. Though Cyclosporine A was once promising, the large CIRCUS trial (2015) phase III randomized trial ≈970 randomized; evaluable at 1 year: cyc: 59.0% versus control 58.1%; odds ratio 1.04 (95% CI 0.78–1.39); *p* = 0.77. According to the experiment, administering IV cyclosporine prior to PCI did not prevent unfavorable cardiac remodeling or enhance clinical outcomes when compared to a placebo [105]. Thus, CsA did not improve clinical outcomes or prevent adverse remodeling in the CIRCUS trial.

Levosimendan: A calcium-sensitizing inotrope that also opens mitochondrial ATP-dependent potassium channels. A 2021 meta-analysis of eight AMI trials (*n* = 951) found that levosimendan (typically given in cardiogenic shock complicating MI) was associated with *reduced acute and 1-year mortality* [106]. Its benefit is thought to derive from improved cardiac function plus cardioprotection via K^+^ATP opening (which reduces calcium overload and apoptosis).

Studies also suggest that although nitric oxide (NO) and nitrite showed early promise for reducing reperfusion injury in STEMI, larger trials have confirmed consistent benefits only in certain subgroups, suggesting more research is needed [44]. In the NIAMI trial (Siddiqi et al. [107]), 229 patients were enrolled—118 received sodium nitrite and 111 received a placebo. Early CMR (6–8 days) showed median infarct size 22% (nitrite) versus 20% (placebo); *p* = 0.30. In the 6-month CMR subgroup (*n* = 118), final infarct size was median 12.0% (nitrite) versus 14.0% (placebo); effect −1.7% (95% CI −3.2 to +5.5); *p* = 0.19. There were no significant differences in LV volumes, LVEF, or biomarker (troponin-I/CK) AUCs. Safety events were similar; deaths were 1 in the nitrite arm versus 4 in placebo. Thus, intravenous sodium nitrite given immediately before reperfusion did not reduce infarct size [107].

In contrast, many other tested therapies have been disappointing in clinical trials. Volatile anesthetics (e.g., sevoflurane sedation during PCI) did *not* reduce infarct size or improve LV function in a randomized STEMI trial. There was no difference in SPECT LVEF at 6 months (51.7% vs. 51.0%; mean difference 0.7%, 95% CI −5.9 to 7.3; *P* = 0.831) or echocardiographic LVEF at 1 year (54.8% vs. 53.9%; *P* = 0.716) between the SIAMI pilot and its one-year substudy (*N* = 46 completing follow-up). The authors attributed the neutral result to a smaller sample size, a lower-risk population, short/low-dose sevoflurane exposure (30 min, MAC ~ 0.5), possible confounding (such as opioid use), and limited imaging sensitivity [108].

Even early aldosterone antagonist therapy (e.g., spironolactone) given post-MI did not improve outcomes in patients without heart failure. This was seen in the ALBATROSS randomized trial (*n* = 1603) where an early MRA regimen (IV potassium canrenoate 200 mg then oral spironolactone 25 mg once daily for 6 months) did not reduce the 6-month composite of death, resuscitated cardiac arrest, serious ventricular arrhythmia, ICD indication or new/worsening heart failure (11.8% vs. 12.2%; HR 0.97, 95% CI 0.73–1.28), and treatment increased hyperkalemia (>5.5 mmol/L) (3.0% vs. 0.2%; *p* < 0.0001); an exploratory subgroup analysis suggested lower mortality in STEMI patients (0.5% vs. 2.4%; HR 0.20) but this was not a prespecified primary finding and requires confirmation [109].

According to Beygui et al. [109], the trial's neutral overall result is likely due to a number of factors, including the potential heterogeneity between STEMI and NSTEMI (the observed STEMI signal was exploratory), potential regimen/timing issues, a clear increase in hyperkalemia with treatment that offsets net benefit, and the enrollment of a lower-risk MI population without established heart failure (yielding low-event rates and reducing power to detect benefit) (JACC 2016). Table 4 summarizes key human clinical trials and meta-analyses of pharmacological strategies to prevent myocardial reperfusion injury in STEMI.

**Table 4:** Pharmacological strategies to mitigate myocardial reperfusion injury in STEMI

Intervention/trial (year)/study type	Drug/intervention	Comparator		Sample size (*N*)	Major numerical outcomes	Status	Design limitations/likely reasons for neutral/failed result	Potential alternatives/next steps	Reference
N-acetylcysteine (NACIAM—Pasupathy et al. 2017)—double-blind RCT	High-dose IV NAC + nitrate early with PCI	Nitrate alone/placebo		*N* = 112 (randomized)	CMR infarct size median 11.0% (NAC) versus 16.5% (placebo); *P* = 0.02. Myocardial salvage index doubled in NAC arm	Positive in this RCT but needs confirmation	Small RCT(s); modest CMR subset sizes; requires confirmation	Larger RCTs or confirmatory trials in enriched populations	[86]
Intracoronary NAC (Nozari et al. 2018)—RCT	Intracoronary N-acetylcysteine administered at PCI	Placebo/standard care		*N* = 100	TIMI grade-3 flow 94% (NAC) versus 80% (placebo), *p* = 0.03; accelerated troponin decline	Positive for reperfusion markers in this trial	Single-center, modest sample size	Larger trials or multicenter replication	[87]
High-dose Vitamin C (meta-analysis)—pooled RCTs	IV ascorbate prior to PCI (various trials pooled)	Control		Pooled *N* = 1185	Lowered cardiac injury biomarkers and inflammatory markers; imaging/clinical benefits inconsistent	Biomarker improvements only	Heterogeneous trials pooled; biomarker improvements not consistently translating to clinical endpoints	Combine antioxidant strategies with other cardioprotective measures; larger outcome trials	[88]
Dapagliflozin (DAPA-MI—2023)—large RCT	SGLT2 inhibitor (dapagliflozin) started soon after MI versus placebo in patients without diabetes	Placebo		*N* = 4017	Did not reduce combined endpoint of death or Heart failure at 1 year (HR ≈ 0.95; 95% CI 0.64–1.40); improved cardiometabolic composite (per draft)	Neutral for death/HF at 1 year; metabolic benefits	Outcome focused; not designed for early remodeling/infarct size effect	Further mechanistic studies or different endpoints/timing	[89]
Trimetazidine (meta-analysis of 14 RCTs)	Pre-PCI trimetazidine versus control (various RCTs pooled)	Control		Pooled across 14 RCTs	Reduced periprocedural troponin-I release and improved ejection fraction	Positive pooled signal for periprocedural injury reduction	Heterogeneous trial designs; variable endpoints	Larger, standardized RCTs to confirm effect on infarct size/clinical endpoints	[90]
Adenosine (meta-analysis, 2024)—pooled RCTs	Adjunctive adenosine (intracoronary or IV) during PCI	Control		Pooled *N* = 2467	Increased ST-segment resolution (RR ≈ 1.30; *P* < 0.001); reduced no-reflow (RR ≈ 0.35; *P* < 0.001); lower MACCE (RR ≈ 0.67; *P* = 0.003); reduced new HF (RR ≈ 0.66; *P* = 0.044)	Positive pooled effects on reperfusion parameters and clinical endpoints	Meta-analysis of heterogeneous RCTs; protocol variation across studies	Consider intracoronary/IV adenosine in selected patients; further RCTs with standardized protocols	[92]
Nicorandil (ChangE 2022)—randomized	IV nicorandil (6 mg bolus + infusion) immediately before PCI	Placebo		*N* = 238	Infarct size at 1 week (CMR): 26.5 ± 17.1 g versus 32.4 ± 19.3 g; *P* = 0.022. No-reflow 9.2% versus 26.3% (*P* = 0.001). ST-resolution 90.8% versus 78.0% (*P* = 0.006). LVEF 6 days: 47.0% versus 43.3% (*P* = 0.011); 6 months: 50.1% versus 46.4% (*P* = 0.009)	Positive (reduced infarct size and no-reflow)	Trial size/phase not fully detailed in draft; needs replication	Replicate in larger/multicenter RCTs; consider patient selection	[93]
Pentoxifylline (PENTOS-PCI, Kakavand et al. 2023)—RCT	IV pentoxifylline before PCI	Placebo		*N* = 161	TIMI-3 flow 71.3% (pentoxifylline) versus 66.3% (placebo), *p* = 0.40; MBG3 87.5% versus 85.2% (*p* = 0.79); corrected TIMI frame count similar	Neutral (no benefit)	Modest sample size; angiographic endpoints only	Trial neutral; further research not supported by this result	[94]
Colchicine (COVERT-MI 2021)—double-blind RCT	Oral colchicine loading dose at reperfusion	Placebo		*N* = 192	Infarct size (MRI at 5 days) median ≈26 g (colchicine) versus ≈28 g (placebo); *P* = 0.87. GI side effects 34% versus 11% (*p* = 0.0002)	Neutral (no reduction in infarct size) and more AEs	Moderate-size RCT; safety signals (GI)	Not supported for perireperfusion cardioprotection	[95]
Tocilizumab (ASSAIL-MI, 2021)—RCT	Single IV dose of IL-6 receptor inhibitor before PCI	Placebo		*N* = 199 (101 to tocilizumab, 98 to placebo)	Myocardial salvage index 69.3 ± 19.3% versus 63.6 ± 20.8% (adj diff 5.6 percentage points; *P* = 0.04). Final infarct size 7.2% versus 9.1% (*P* = 0.08)	Positive for salvage index; infarct size reduction not significant	Sample size/details not fully given in draft; final infarct size not statistically significant	Promising mechanistic signal—requires larger confirmatory outcome trials	[96]
β-blockers/Metoprolol (METOCARD-CNIC; EARLY-BAMI)—RCTs/meta	IV metoprolol before reperfusion (various dosing/timing)	Control	METOCARD-CNIC *N* = 270; EARLY-BAMI *N* = 683		METOCARD-CNIC: early MRI ~ 20% smaller infarct; 6-month LVEF 48.7% versus 45.0% (adj effect 3.49%, *p* = 0.025); severe LV dysfunction 11% versus 27% (*p* = 0.006). EARLY-BAMI: CMR infarct size 15.3% versus 14.9% (*p* = 0.616)	Mixed results: METOCARD positive; EARLY-BAMI neutral	Variation in timing/dose; later/lower-dose neutral	Standardize timing/dose; target early prereperfusion administration	[103] [104]
Cyclosporine A (CIRCUS, 2015)—phase III RCT	IV cyclosporine before PCI	Placebo		*N* ≈ 970 randomized	At 1 year: cyc 59.0% versus control 58.1%; OR 1.04 (95% CI 0.78–1.39); *p* = 0.77	Failed to improve clinical outcomes or prevent remodeling—not effective in humans	Phase III negative; biological target not sufficient in humans	No further development for this indication	[105]
Levosimendan (meta-analysis 2021)—pooled placebo-controlled AMI trials (8 RCTs)	Levosimendan IV; in AMI/cardiogenic shock settings	Placebo/standard care (varied across trials)		*N* = 951 (pooled across 8 trials)	Associated with reduced acute and 1-year mortality in pooled analysis	Positive signal in pooled AMI data	Heterogeneous trials (different patient mixes, dosing regimens, endpoints); mainly used in cardiogenic shock settings	Consider targeted use, adequately powered RCTs in AMI patients with cardiogenic shock (standardized dosing/timing)	[106]
Sodium nitrite (NIAMI, 2014)—RCT	IV sodium nitrite immediately before reperfusion	Placebo		*N* = 229 (118 nitrite, 111 placebo)	Early CMR 22% versus 20% (*p* = 0.30). 6-month subgroup (*n* = 118) final infarct 12.0% versus 14.0% (effect −1.7%; 95% CI −3.2 to +5.5; *p* = 0.19)	No significant infarct size reduction; neutral	Neutral	Evaluate alternative NO/microvascular strategies; adenosine adjunct; nitrate-based combination regimens, and test in enriched high-risk STEMI cohorts or combined mechanical + pharmacologic protocols	[107]
Volatile anesthetic (SIAMI pilot & 1-yr substudy)—randomized pilot	Sevoflurane sedation during PCI (short exposure 30 min, MAC ~ 0.5)	Standard care		*N* = 46 completed 1-year follow-up (substudy)	SPECT LVEF at 6 months 51.7% versus 51.0% (mean diff 0.7%, 95% CI −5.9 to 7.3; *P* = 0.831); 1-year echo LVEF 54.8% versus 53.9% (*P* = 0.716)	Neutral (pilot/underpowered)	Pilot, small sample, low-risk population, short/low-dose exposure, potential confounders	Larger, adequately powered trials or higher-dose/longer exposure protocols; control confounders	[108]
Early aldosterone antagonist (ALBATROSS, JACC 2016)—randomized	IV potassium canrenoate 200 mg then oral spironolactone 25 mg daily for 6 months	Usual care		*N* = 1603	6-month composite 11.8% versus 12.2% (HR 0.97; 95% CI 0.73–1.28). Hyperkalemia (>5.5 mmol/L) 3.0% versus 0.2% (*p* < 0.0001). Exploratory STEMI subgroup mortality 0.5% versus 2.4% (HR 0.20)	Neutral overall; safety offset; STEMI exploratory signal requires confirmation	Heterogeneous MI population; low-event rates; safety (hyperkalemia) offset	Enrich future trials for STEMI/high-risk patients (reduced LVEF or HF), optimize timing/regimen (dose, IV vs. oral), implement strict K^+^/renal monitoring and exclusion criteria to reduce hyperkalemia, and power for clinical endpoints rather than low-event mixed MI cohorts	[109]

Summary of key human randomized trials and meta-analyses of pharmacological strategies to prevent myocardial reperfusion injury. Columns report intervention/comparator details, sample size, principal numerical outcomes, trial status, design limitations/reasons for neutral/failed results, suggested next steps, and reference. Only human trials and pooled human analyses cited in the submitted manuscript are included. PCI = percutaneous coronary intervention; PPCI = primary PCI; STEMI = ST-elevation myocardial infarction; MI = myocardial infarction; AMI = acute myocardial infarction; CMR = cardiac magnetic resonance; IS = infarct size; TIMI = thrombolysis In Myocardial Infarction; MBG = myocardial blush grade; SPECT = single-photon emission computed tomography; LVEF = left ventricular ejection fraction; HF = heart failure; MACCE/MACE = major adverse cardiac (and cerebrovascular) events; HR = hazard ratio; OR = odds ratio; CI = confidence interval; LV = left ventricular; IS = infarct size; MaR = myocardium at risk; IS/MaR = infarct size expressed as a proportion of myocardium at risk; MSI = myocardial salvage index (proportion of at-risk myocardium salvaged after reperfusion); MVO = microvascular obstruction; IMH = intramyocardial hemorrhage; TIMI = thrombolysis in myocardial infarction; cTFC = corrected TIMI frame count; MBG/MBG3 = myocardial blush grade; RR = relative risk; SGLT2 = sodium-glucose cotransporter-2; MAC = minimum alveolar concentration; adj = adjusted; adj diff = adjusted difference; echo = echocardiography; NAC = N-acetylcysteine

In summary, reperfusion injury is a complex process. Studies emphasizing combined strategies are under investigation. No single “magic bullet” has been found, but multimodal therapy shows promise.

## Future directions and research gaps

Reperfusion damage continues to be a major cause of morbidity and mortality even after PPCI has proven effective in treating STEMI [110]. Recent studies have identified several areas for further investigation.

### Mitochondrial dynamics and genetic targets

Reperfusion injury may be caused by mitochondrial gene alterations that trigger cell death [111, 112]. It is influenced by gene-related factors such as cyclophilin D (CyPD), dynamin-related protein 1 (Drp1), and members of the NADPH oxidase (NOX) family. Studies on the *NOX gene* have produced conflicting results. Two additional genes, *Augmenter of liver regeneration (ALR)* and *Optic Atrophy 1 (OPA1),* may also be potential contributors to mitochondrial gene-induced reperfusion damage, according to a recent study [111].

The relationship between IRI and the current gene variations for *aldehyde dehydrogenase (ALDH2), BCL2 Interacting Protein 3 (BNIP3), and OPA1* in various organs and situations needs more investigation. Mendelian randomization studies with validated biomarkers are required to confirm the treatment potential of these mitochondrial genes to establish a causal association between one of these genes and IRI [111].

### Emerging biomarkers for prognosis and risk stratification

Although cardiac troponins remain the most accurate method of diagnosing myocardial infarction, additional biomarkers have demonstrated potential in risk assessment and prognosis [113].

In addition to being a multipurpose soluble pattern recognition molecule that is essential for innate immunity and the inflammatory response, pentraxin 3 (PTX3) is now known to be a distinct marker of cardiovascular disease [114]. Cardiomyocytes, endothelial cells, and fibroblasts all express ST2, a member of the interleukin-1 receptor family [115]. Future treatment of individuals with STEMI may involve the use of pentraxin and sST2 in addition to NT-proBNP [113].

Endothelial cells in coronary arteries release lipocalin-type prostaglandin D synthase (L-PGDS) into the bloodstream, which has been identified as a possible biomarker of coronary circulation especially during angina episodes. Because L-PGDS rises in the bloodstream more quickly than other biomarkers, it may be a useful early diagnostic in the setting of STEMI. It is a promising option for risk assessment and early detection due to its excellent sensitivity and specificity for coronary ischemia, which are on par with recognized indicators [116]. Other emerging markers include Galectin-3 and high-sensitivity C-reactive protein (hs-CRP), reflecting fibrosis/inflammation and systemic inflammation, respectively. Galectin-3 concentrations have been linked to adverse cardiac outcomes and prognosis [117], and hs-CRP provides prognostic information beyond troponin [118]. Combined biomarker strategies (for example, combining sST2 with Galectin-3 or hs-CRP) are being explored to improve risk prediction in STEMI.

### Applications of artificial intelligence in STEMI diagnosis and risk prediction

Artificial intelligence-enhanced ECGs reduce the time from ECG acquisition to balloon inflation and the total door-to-balloon time by facilitating faster STEMI detection [119]. Advanced diagnostic techniques of artificial intelligence are applied for the early diagnosis of STEMI. A retrospective observational study used machine learning models to cluster participants based on lipid profile abnormalities such as elevated lipoprotein (a), decreased high-density lipoprotein cholesterol (HDL-C), and apolipoprotein A1, which are common causes of heart disease. The study found lipid abnormalities, helped identify patients at higher risk for STEMI, and allowed for patient risk assessment [120, 121]. Artificial intelligence is more precise in myocardial tissue analysis than conventional techniques as it has facilitated myocardial segmentation on computed tomography scans along with the analysis of degrees of muscle change during the diastolic and systolic phases [121].

### Challenges and considerations

Despite all of artificial intelligence's benefits, issues still persist. For instance, hypertrophied ventricles could be mistakenly identified as fibrotic tissue by artificial intelligence algorithms. This could result in inaccurate diagnoses [121]. These algorithms necessitate large amounts of patient data, which raises privacy and consent concerns. Additionally, they may exhibit algorithmic bias, which could cause them to behave differently depending on the demographic group. Regulations are still catching up. For example, the FDA has approved several AI-based ECG interpretation tools based on historical data, but there are not many prospective studies demonstrating actual patient benefits. Uncertainty around who is in charge of AI-driven choices and the absence of standardized approval procedures (from agencies like the FDA or EMA) continue to be significant obstacles. Transparency, external validation and clinician oversight will be needed to safely integrate AI into STEMI care [122].

Regulatory and ethical concerns are also crucial. Everyone should have access to new diagnostic resources and therapies in order to stop the current health disparities from getting worse [122]. Similarly, new gene-editing treatments, including those based on clustered regularly interspaced short palindromic repeats (CRISPR), bring up significant issues with long-term safety, informed consent, and germline editing [123].

### Addressing sex-specific risk factors

There are sex-specific risk factors for in-hospital mortality among patients with acute coronary syndrome, according to recent research employing machine learning techniques. For example, acute renal failure and elevated troponin T levels were more predictive in men, while chronic kidney failure and tachycardia were important predictors in women. In order to create individualized treatment plans, it is essential to acknowledge and address these variations [124].

Clinical differences by sex have practical implications. Women with STEMI often present at older ages with more comorbidities and different biomarker profiles (e.g., higher NT-proBNP levels) [125]. Many ACS trials historically under-enroll women, limiting sex-specific evidence. As a result, policies (like NIH’s mandate to consider sex as a biological variable) now encourage balanced representation. Women generally have worse outcomes after STEMI and are less likely to receive guideline-directed therapy. To assist reduce these inequalities, targeted strategies have been proposed. These include risk calculators that take sex variations into account and cardiac rehabilitation programs tailored to women [126]. AI-based risk should also be trained and evaluated on datasets that include a fair mix of men and women, to prevent bias and make sure they are accurate for anyone. Collectively, these developments increase our understanding of the mechanisms behind reperfusion injury and open the door to more early and individualized therapies in the therapy of STEMI.

## Conclusion

Reperfusion therapy in STEMI is a lifesaving, yet paradoxical intervention that is capable of simultaneously inducing myocardial injury through oxidative stress, calcium overload, and opening of the mitochondrial permeability transition pore (mPTP) and programmed cell death. Clinically, they manifest as no-reflow, stunning, arrhythmias, hemorrhage, and finally, lethal injury. These consequences are independently linked to larger infarcts, poor remodeling, heart failure, and higher mortality. Despite improvements in early identification (sST2 biomarkers, cardiac MRI), no treatments have shown consistent efficacy in reducing reperfusion injury. Mechanical and pharmacological methods indicate cardioprotection, but their translation to routine care is challenging. No single solution has been identified. Contemporary evidence favors a multimodal strategy (combined mechanical conditioning, targeted pharmacology, biomarker-guided enrollment, and AI-assisted triage) as the most plausible route to clinically meaningful cardioprotection. Finally, safe deployment of AI, mitigation of algorithmic bias, and concerted efforts to address sex-specific gaps (trial enrollment and sex-aware risk models) must accompany mechanistic/therapeutic advances to ensure equitable benefit. Bridging this gap is important for optimization of myocardial salvage and extension of long-term survival beyond what is currently achieved with revascularization alone. After all, reperfusion injury is still a double-edged sword that has researchers and clinicians stumped, underscoring the urgent need for novel treatments to shift the scales in favor of myocardial preservation and favorable outcomes.

## Data Availability

No datasets were generated or analysed during the current study.

## Abbreviations

ACS: Acute coronary syndromes
AMI: Acute myocardial infarction
AUC: Area under the ROC curve
CARIOCA: Combined application of remote and intracoronary ischemic conditioning in acute myocardial infarction
CCTA: Coronary CT angiography
CK-MB: Creatine kinase-MB
CI: Confidence interval
CMR: Cardiac magnetic resonance (cardiac MRI)
CsA: Cyclosporine A
ECG: Electrocardiogram
Gal-3: Galectin-3
HF: Heart failure
HDL-C: High-density lipoprotein cholesterol
hs-CRP/hsCRP: High-sensitivity C-reactive protein
hs-cTnT: High-sensitivity cardiac troponin T
IMH: Intramyocardial hemorrhage
IPoC: Ischemic postconditioning
IRI: Ischemia-reperfusion injury
IS: Infarct size
LV: Left ventricle/left ventricular
LVEDV: Left ventricular end-diastolic volume
LVEF: Left ventricular ejection fraction
MACE/MACCE: Major adverse cardiovascular events/major adverse cardiac and cerebrovascular events (used in the manuscript; both acronyms appear)
MaR: Myocardium at risk
MIRI: Myocardial infarction reperfusion injury
MVO: Microvascular obstruction
NAC: N-acetylcysteine
NT-proBNP: N-terminal pro-B-type natriuretic peptide
OR: Odds ratio
PCI: Percutaneous coronary intervention
PPCI: Primary percutaneous coronary intervention
RIC: Remote ischemic conditioning
ROC: Receiver operating characteristic
RCT: Randomized controlled trial
RR: Relative risk
ROS: Reactive oxygen species
sST2: Soluble ST2
SPECT: Single-photon emission computed tomography
STEMI: ST-elevation/ST-segment elevation myocardial infarction
Tc-99m: Technetium-99m
TIMI: Thrombolysis in myocardial infarction
